# Successful radical nephrectomy for renal cell carcinoma in a pregnant patient: a case report and literature review

**DOI:** 10.1097/MS9.0000000000004223

**Published:** 2025-10-28

**Authors:** Anas R. Tuqan, Ihab Hemieid, Eman A.S. Omari, Anas M. Barabrah, Omar E. Salah, Tariq Asi

**Affiliations:** Faculty of Medicine, Al-Quds University – School of Medicine, Abu-Dis, East Jerusalem, Palestine

**Keywords:** cancer, case report, nephrectomy, pregnancy, RCC

## Abstract

**Introduction and importance:**

Renal cell carcinoma (RCC) in pregnancy is extremely rare, with an estimated incidence of 13 per million pregnancies. Papillary RCC, a less common subtype, poses unique diagnostic and therapeutic challenges during gestation.

**Case presentation:**

A 38-year-old woman at 26 weeks’ gestation presented with persistent right flank pain unresponsive to initial treatment. Imaging via ultrasound and magnetic resonance imaging (MRI) revealed a large right renal mass. Following multidisciplinary evaluation, an open radical nephrectomy was performed. Histopathology confirmed papillary RCC, pT2, Grade III. Both maternal and fetal outcomes were favorable, with no recurrence at 7 months’ follow-up.

**Clinical discussion:**

Diagnosing RCC during pregnancy is challenging due to symptom overlap with common gestational disorders such as urinary tract infections or preeclampsia. Ultrasound and MRI, which avoid ionizing radiation, are the preferred imaging modalities, but can be limited by anatomical changes during pregnancy. Surgical resection remains the definitive treatment, with the second trimester offering a balance between minimizing fetal risk and preventing tumor progression. Decisions on timing and surgical approach must be individualized, considering tumor size, location, and gestational age. A multidisciplinary team involving urology, obstetrics, anesthesiology, and neonatology is essential for optimal maternal–fetal outcomes. Despite the predominance of clear cell RCC in pregnancy, this case highlights the need for awareness of rarer subtypes like papillary RCC. The absence of standardized management guidelines underscores the importance of case-by-case assessment.

**Conclusion:**

Timely diagnosis, individualized surgical planning, and coordinated multidisciplinary care are crucial in managing RCC during pregnancy, particularly for rare histological variants.

## Introduction

Renal cell carcinoma (RCC) is a rare condition encountered during pregnancy, with only approximately 100 cases reported in the literature^[1]^. Despite its rarity, RCC is the most common urological malignancy reported in pregnant women, accounting for half of all primary renal tumors during gestation^[2,3]^. The incidence of cancer during pregnancy is estimated to be about 1 in 1000 pregnancies, with breast cancer, melanoma, cervical cancer, and lymphomas being more frequently diagnosed^[3]^. RCC typically represents about 3% of all adult malignancies, with a male predominance in the general population^[4]^. However, in pregnant women, the diagnosis can be challenging due to symptoms that mimic common pregnancy-related disorders, such as urinary tract infections or preeclampsia.

**HIGHLIGHTS**

- Renal cell carcinoma in pregnancy is rare but serious, requiring careful diagnosis as symptoms mimic common pregnancy issues.
- Ultrasound and magnetic resonance imaging are safest for diagnosis, while CT should be used cautiously.
- Surgery timing is critical, with the second trimester often ideal.

The management of RCC in pregnant women is complex due to the lack of specific guidelines, and treatment often involves surgical resection, either open or laparoscopic radical nephrectomy, to ensure both maternal and fetal well-being^[4]^. This case report aims to highlight the challenges and considerations involved in diagnosing and treating RCC in pregnant patients.

This case has been reported in line with the SCARE 2025 criteria^[5]^.

## Case presentation

A 38-year-old primigravida woman at 26 weeks of gestation, with a body mass index of 26, no known comorbidities, and a nonsmoking lifestyle, presented with mild right flank pain. Her pregnancy had otherwise been uncomplicated. On clinical examination, she was afebrile and hemodynamically stable. Abdominal examination revealed mild right flank tenderness without peritoneal signs.

Initial laboratory investigations, including complete blood count, renal function tests, and urinalysis, were within normal limits. In particular, the urinalysis showed no signs of urinary tract infection, excluding an initial working diagnosis of pyelonephritis. Differential diagnoses at presentation included urinary tract infection, physiological hydronephrosis of pregnancy, and, less likely, other intra-abdominal causes such as cholecystitis.

**Figure 1.**
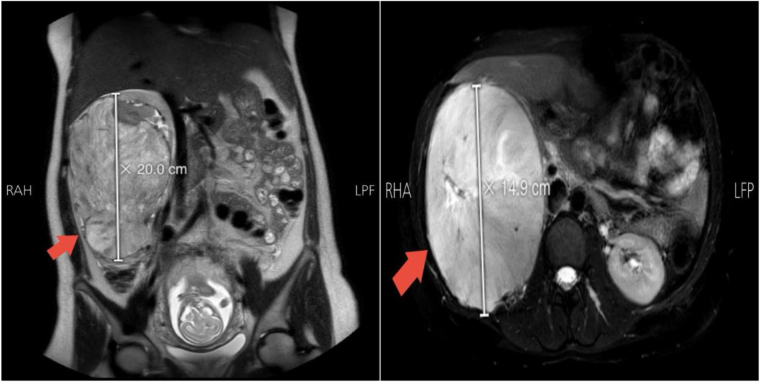
MRI showing a large right renal mass measuring approximately 20.0 cm in height (left) and 14.9 cm in width (right).

Given the persistence of symptoms, an abdominal ultrasound was performed. This revealed a large, hypervascular right renal mass with no features of hydronephrosis. The lesion was initially suspected to be a renal angiomyolipoma; however, the absence of macroscopic fat on imaging raised suspicion of an alternative pathology. To further evaluate the lesion, a non-contrast magnetic resonance imaging (MRI) scan was obtained, which confirmed a large right renal mass measuring approximately 20 cm in diameter (Fig. 1). The differential diagnosis at this stage included RCC, oncocytoma, or angiomyolipoma.

The obstetrics team was consulted, and a detailed fetal examination and ultrasound were performed, demonstrating a normal fetus with no evidence of compromise. Fetal monitoring was continued throughout the perioperative period.

Given the size, vascularity, and malignant potential of the lesion, a multidisciplinary team comprising urology, obstetrics, anesthesiology, and neonatology decided to proceed with surgical intervention during pregnancy. One week after her initial presentation, at 26 weeks of gestation, the patient underwent an open right radical nephrectomy via a right subcostal transabdominal incision. The procedure lasted 1 hour and 20 minutes, with an estimated blood loss of 10–20 mL. General anesthesia was used, and intraoperative fetal monitoring was performed. Thromboprophylaxis with low molecular weight heparin and compression stockings was initiated. A partial nephrectomy was not feasible due to the large size of the tumor (20 cm) and its infiltrative involvement of the kidney; thus, radical nephrectomy was chosen.

Histopathological analysis of the resected specimen (20 cm in size) confirmed papillary type RCC, classified as pT2, Grade III, with central necrosis and negative surgical margins.

The patient’s postoperative course was uneventful. Obstetric follow-up revealed normal fetal growth, with repeated ultrasounds confirming no complications. At 39 weeks of gestation, she delivered a healthy infant vaginally, with no evidence of perinatal distress or abnormality. At 7 months postoperative follow-up, the patient remains well, with no clinical or radiological evidence of tumor recurrence.

## Discussion

RCC during pregnancy is a rare occurrence, with only around 100 cases reported in the literature^[1]^. Among these, papillary RCC is even less common, with the vast majority of cases involving the clear cell subtype^[6]^. Our case contributes to this limited pool by presenting a large, papillary RCC diagnosed at 26 weeks of gestation and managed successfully through open radical nephrectomy during pregnancy. This histologic variant remains underreported, and its presentation during gestation adds further diagnostic and therapeutic complexity.

Pregnancy-related physiological changes, including increased renal blood flow, hormonal shifts, and immune modulation, may influence tumor behavior, although the exact impact on RCC biology remains uncertain^[7,8]^. In our patient, the presentation with isolated right flank pain, absence of hematuria or systemic symptoms, and normal laboratory findings underscores the difficulty of distinguishing RCC from more common gestational conditions like urinary tract infection, hydronephrosis, or renal stones^[1]^.

Imaging plays a pivotal role in diagnosis; while ultrasound is typically first-line due to safety and accessibility, its diagnostic capacity may be limited in later pregnancy due to uterine size and patient body habitus.^[9,10]^. MRI is commonly used for staging and evaluating the treatment of kidney cancer. It offers high-resolution images without the need for intravenous contrast and is safe during pregnancy because it does not involve ionizing radiation. This makes MRI a valuable tool for diagnosis and treatment planning in pregnant patients, helping to prevent misdiagnosis and delays.^[10]^ In our case, MRI was instrumental in defining the lesion characteristics and guiding management, avoiding fetal exposure to ionizing radiation.

Treatment decisions for RCC during pregnancy remain highly personalized due to the lack of standardized guidelines^[11]^. Surgery should account for factors like minimizing teratogenic risks and avoiding pregnancy disruption, including the choice of surgical procedure, approach, timing, and positioning. The best timing for surgery depends on the tumor’s behavior and neonatal survival rates at different gestational stages. RCC grows at approximately 0.4 cm annually, with a tumor volume doubling time of around 500 days.^[12]^ If RCC is diagnosed during the first trimester, surgery should be performed without delay despite the potential risks of miscarriage or congenital abnormalities.^[11]^ Termination may be considered before surgery due to the risk of metastasis.^[13]^ The optimal timing of surgery in the second trimester is debated; the current literature emphasizes the need for a personalized approach to treatment. Some experts advise against surgery during this period because of risks such as uterine contractions and fetal distress^[14]^, while others advocate for it because of increased risks later in pregnancy.^[15]^ In the third trimester, surgery can be performed with a cesarean section or postponed until after vaginal delivery, with close monitoring of the tumor and active surveillance for rapid growth.^[14]^ Once the surgical timing is established, the appropriate nephrectomy approach should be selected based on tumor size. Partial nephrectomy is preferred for smaller T1 tumors, while radical nephrectomy is usually recommended for T2 and larger tumors.^[16]^ These procedures can be performed via open, laparoscopic, or robotic techniques. Laparoscopy offers benefits like quicker recovery and less pain, but carries risks of direct trauma to the fetus and uterus. Safe laparoscopic access can be achieved using the Hasson technique, with trocar and port placement adjusted according to uterine size.^[11,17]^. However, given the tumor’s large size (20 cm), vascularity, and risk of malignant progression, delaying surgery posed significant dangers. Partial nephrectomy was not feasible, and the decision to perform an open radical nephrectomy during the second trimester, generally considered the safest time for non-obstetric surgery, was made through a multidisciplinary consensus. The open approach was chosen due to the tumor’s size and complexity, and the need to ensure cancer removal in a controlled setting^[11,15,17]^. The procedure was completed without intraoperative or postoperative complications, and fetal development was unaffected.

The strengths of this case include the rarity of the histological subtype, the papillary RCC, the use of safe and effective imaging during pregnancy, and successful management through a coordinated multidisciplinary approach involving urology, obstetrics, anesthesiology, and neonatology. The positive outcome, with a full-term vaginal delivery of a healthy infant and no evidence of recurrence at 7 months, reinforces the safety and effectiveness of timely surgical intervention when necessary. However, limitations should be acknowledged: the follow-up period is relatively short, and long-term oncological outcomes remain uncertain; the absence of histopathological images limits diagnostic detail; and as a single-case report, its generalizability is limited. Including detailed histological and immunohistochemical data in future reports would strengthen the evidence base for managing RCC during pregnancy.

## Conclusion

RCC during pregnancy is an infrequent diagnosis, with an incidence of approximately 13 per million pregnancies. This case of papillary RCC at 26 weeks’ gestation demonstrates the critical role of timely imaging, particularly ultrasound, MRI, and multidisciplinary coordination in achieving optimal outcomes. Surgical resection remains the mainstay of treatment, with second-trimester intervention often considered the safest window. This report highlights the importance of individualized, evidence-informed management in the absence of standardized guidelines.

## Data Availability

Not applicable.

## References

[1] Boussios S Pavlidis N. Renal cell carcinoma in pregnancy: a rare coexistence. Clin Transl Oncol 2014;16:122–27. 24002946 10.1007/s12094-013-1105-2

[2] Gladman MA MacDonald D Webster JJ. Renal cell carcinoma in pregnancy. J R Soc Med 2002;95:199–201. 11934912 10.1258/jrsm.95.4.199 PMC1279516

[3] Pavlidis N. Cancer and pregnancy: what should we know about the management with systemic treatment of pregnant women with cancer? Eur J Cancer 2011;47:S348–S352. 21944011 10.1016/S0959-8049(11)70199-X

[4] Xu H Tan S. Diagnosis and treatment of renal cell carcinoma during pregnancy. Cancer Manag Res 2021;13:9423–28. 35002320 10.2147/CMAR.S345309 PMC8721013

[5] Kerwan A Al-Jabir A Mathew G. Revised Surgical CAse REport (SCARE) guideline: an update for the age of Artificial Intelligence. Prem J Sci 2025;10:100079

[6] Ferreira B Alves AR, Rodrigues AC. Renal cell carcinoma in pregnancy: a case report of a rare diagnosis. Cureus 2025;17:e76960. 39906473 10.7759/cureus.76960 PMC11793927

[7] Chaliha C Stanton SL. Urological problems in pregnancy. BJU Int 2002;89:469–76. 11929469 10.1046/j.1464-410x.2002.02657.x

[8] McGladdery SL Aparicio S Verrier-Jones K. Outcome of pregnancy in an Oxford-Cardiff cohort of women with previous bacteriuria. Q J Med 1992;83:533–39. 1484930

[9] Vandecaveye V Amant F Lecouvet F. Imaging modalities in pregnant cancer patients. Int J Gynecol Cancer 2021;31:423–31. 33649009 10.1136/ijgc-2020-001779 PMC7925814

[10] Committee Opinion No. 723: guidelines for diagnostic imaging during pregnancy and lactation. Obstet Gynecol 2017;130:e210–e216. 28937575 10.1097/AOG.0000000000002355

[11] Caglayan A Rabbani RD Sanchez E. Gestational renal cell cancer – an update. Anticancer Res 2023;43:3871–80. 37648307 10.21873/anticanres.16574

[12] Chawla SN Crispen PL Hanlon AL. The natural history of observed enhancing renal masses: meta-analysis and review of the world literature. J Urol 2006;175:425–31. 16406965 10.1016/S0022-5347(05)00148-5

[13] Zhao Y Yang Z Xu W. Management of renal tumors during pregnancy: case reports. BMC Nephrol. 2021;22:127. 33836679 10.1186/s12882-021-02318-w PMC8035726

[14] Berczi C Flasko T. Renal tumor in pregnancy: a case report and review of the literature. Urol Int 2015;99:367–69. 26279416 10.1159/000437337

[15] Akpayak IC Shuiabu SI Ofoha CG. Renal cell carcinoma in pregnancy: still a management challenge. Afr J Urol 2015;21:167–70.

[16] Ball E Waters N Cooper N. Evidence-based guideline on laparoscopy in pregnancy: commissioned by the British Society for Gynaecological Endoscopy (BSGE) endorsed by the Royal College of Obstetricians & Gynaecologists (RCOG) [published correction appears in Facts Views Vis Obgyn. 2020;11:261]. Facts Views Vis Obgyn 2019;11:5–25. 31695854 PMC6822954

[17] Dell’Atti L Borghi C Galosi AB. Laparoscopic approach in management of renal cell carcinoma during pregnancy: state of the art. Clin Genitourin Cancer 2019;17:e822–e830. 31227431 10.1016/j.clgc.2019.05.025

